# Health Information Accessed on the Internet: The Development in 5 European Countries

**DOI:** 10.1155/2012/297416

**Published:** 2012-12-05

**Authors:** Per Egil Kummervold, Rolf Wynn

**Affiliations:** ^1^Northern Research Institute, Tromsø, Norway; ^2^Telemedicine Research Group, Department of Clinical Medicine, University of Tromsø, N-9037 Tromsø, Norway; ^3^Division of Addiction and Specialized Psychiatry, University Hospital of North Norway, N-9291 Tromsø, Norway

## Abstract

The aim of this study was to summarize and analyse findings from four prior studies on the use of the Internet as a source of health information in five European countries (Norway, Denmark, Germany, Greece, and Portugal). A cross-study comparison of data was performed. All the studies included fit with a trend of a sharp and continuous growth in the use of the Internet for health information access in the major part of the last decade. Importantly, the Internet has become an important mass media source of health information in northern Europe. While the use of the Internet for health information is somewhat less common in the south European countries, its use is also clearly increasing there. We discuss the advantages of cross-study comparisons of data and methodological challenges. As the use of the Internet for health information is likely to peak in some countries in the near future, new population surveys on health information access should focus more on the details of information that is accessed and which sites that are most used and trusted.

## 1. Background

Most people are, at some point, in need of information about health and illness. Health information may help patients and the public improve health-related decisions, and hopefully, improve health outcomes [1–3]. Before the Internet, people relied on other sources for health information, including newspapers and magazines, books, and information given by their doctors, family, and friends. While these sources are still important, the Internet is rapidly establishing itself as a central source of health information [4, 5]. Several studies have examined the use of the Internet for health information during the last decade. However, few attempts have been made at comparing the outcomes of the various studies. In the present study, we address this gap in the literature by comparing results from four European surveys on the use of the Internet for health information access [6–9]. The main question we wish to address in this study is if the data from different European studies of Internet access for health information in the period 2000–2007 show a consistent pattern.

## 2. Methods

In the present study, we draw on aggregated published data from four major European studies of the use of the Internet for health information: European eHealth Trends [4, 7], Norwegian eHealth Study [6], Eurobarometer [8], and Network Society in Portugal [9–12]. In our study, we have chosen to include only surveys that include the countries where we have data on at least three time points, that is, Norway, Denmark, Germany, Portugal, and Greece. However, some results from studies from other European countries and from countries outside Europe suggest similar trends [13–17]. 

The Norwegian Centre for Integrated Care and Telemedicine was among the first in Europe to survey the use of the Internet for health purposes, and the first national survey was conducted in 2000 [6]. This was done using computer-assisted telephone interviews with a target sample of 1000 respondents. The survey was repeated in 2001 and 2003. In 2005 and 2007, the survey was continued as the (European) eHealth Trends survey, adding six additional countries and a total of 16000 respondents in Denmark, Germany, Greece, Latvia, Poland, and Portugal. Many of the same questions were used, making it easy to do comparisons. All these five eHealth Trend surveys (i.e., Norwegian and European) are included. 

In addition, we include data from the Eurobarometer study [8] and the Network Society in Portugal study [9–12]. In September and October 2002, the European Commission, Health and Consumer Protection Directorate-General, performed wave 58.0 of the standard Eurobarometer. This study had a representative sample from 16 European countries with approximately 1000 respondents in each country and examined the sources for health information [8]. There are, however, to our knowledge, no later reports from Eurobarometer addressing these issues. We also draw on additional data for Portugal. The Network Society in Portugal study [9–12] is a national survey based on a representative sample of 711 persons, conducted in March–June 2003.

## 3. Results

The Norwegian eHealth survey, conducted in 2000, 2001, and 2002, reported Internet for health use rising from 19% in 2000 to 33% in 2002. The (European) eHealth Trends study reported [4, 7] that overall 42.3% of the population in the seven target countries used the Internet for health purposes in 2005. In 2007, this number had increased to 52.2%. In all the individual countries there was an increase in the use of the Internet for this purpose; however, there was also much variation. The south European countries Greece and Portugal had the least use and the two Scandinavian countries had the highest use [7].

The Eurobarometer study [8] found that in 2002, overall, 23.1% of the population in the current 15 EU countries had used the Internet to get information about health. Four of the countries included in the Eurobarometer study had findings relevant to the current study, that is, Denmark (47.2%), Germany (24%), Portugal (14%), and Greece (11.7%). The Network Society in Portugal study found that, in 2003, 19.6% of the population used the Internet for health information retrieval [9–12].

**Figure 1:**
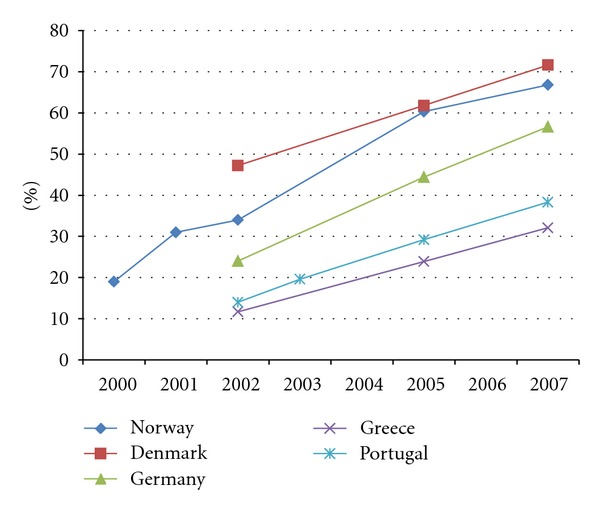
The development of the use of the Internet as a source of health information 2000–2007.

By summarizing the data from the four studies in Table 1 and Figure 1, we are able to make some interesting observations. First, it is clear that in all the five countries included here, there was a steady growth in the use of the Internet for health information in the larger part of the last decade. Second, there were great differences in the use of Internet for health purposes in the European countries we discuss. In general, it was the northern countries (Denmark and Norway) that had the highest usage, trailed by a central European country (Germany), and with the south European countries (Greece and Portugal) with the lowest usage (Figure 1).

## 4. Discussion

Our main finding is that all the included studies fit with a trend of a sharp and continuous growth in the use of the Internet for health information. Projecting from the data from the Norwegian eHealth study and the Norwegian data in the European eHealth Trends study [4, 7], Wangberg et al. [6] stipulated that approximately 80% of Norwegians had become Internet health users by 2010. Thus, in some of the European countries with the highest use of the Internet for health information access, we are likely to see a peak and levelling out of the curve, as the overwhelming majority of those who are able to use and interested in using the Internet for health information access are doing so. Importantly, the Internet has become an important mass media source of health information in northern Europe.

While the use of the Internet for health information has been somewhat less common in the south European countries, its use has also been clearly increasing here. There is no indication that the gap in use between the north European and south European countries has been widening. Rather, it seems as though Greece and Portugal in 2007 had reached the level of use of Norway and Germany in 2002/2003 (cf. Figure 1), and thus one may speculate that the south European countries would catch up in use with the north European countries approximately 5 years after Denmark/Norway have reached their peak levels.

This study does not address the topic of why the reported health information access on the Internet differed between the five countries. However, one obvious underlying factor related to differences between the countries is the access to the Internet in general [4]. The relationship between Internet access in general and the use of the Internet for health information retrieval is of interest and should be explored in future studies. 

Comparing data across different studies does offer advantages as well as methodological challenges. As none of the studies in question (with the exception of the Norwegian eHealth study) contains data from more than two time points, it would have been difficult to see the apparently consistent trends across time and countries without drawing on data from several studies. One potential methodological challenge is that the data of the different studies can be based on different types of samples (i.e., representative versus nonrepresentative samples) or differently formulated questions (i.e., that measure slightly different phenomena). Such differences could potentially distort data, and we cannot rule out if these issues have impacted our findings. The data we have sampled fit quite well with the assumption of a near-linear growth. There was an alleviation of the curve for the Norwegian data in the period 2001-2002, but we consider this to be a very minor deviation from the overall pattern, which possibly could be related to methodological issues (possibly sampling) in the 2001 or 2002 study. Overall, we gather that sampling and question formulation have not been of great importance in the present comparison. 

As the use of the Internet for health information is likely to peak in some countries in the near future, we could possibly have expected to observe a more S-shaped (sigmoid) curve. It should, however, be noted that since most of the data series available to us consist of 3-4 points, such shapes could be difficult to detect. At this point, new population surveys on health information access should focus more on other, but related, issues that are of importance (i.e., to health providers, governments, academics, etc.), such as the details and quality of information that are accessed and which sites that are most used and trusted.

## 5. Conclusions

We have in this study compared findings from four prior studies, covering respondents in five European countries over a substantial part of the last decade. By using this technique, we have been able to demonstrate a trend of steady growth in the use of the Internet for health information access in all five countries. However, as the point of departure differed significantly between the countries, the north European countries appear to be approaching a peak in users while this source of health information has not yet become so central in the south European countries. The importance of the Internet as a source of health information should not be underestimated. As more and more people turn to the Internet for information about health and illness, it becomes essential that health providers, governments, and other parties concerned with public health strive to make efficient use of this tool by providing high-quality information that can be trusted. 

## Figures and Tables

**Table 1:** The use of the internet as a source of health information in five European countries, 2000–2007. Values are reported in percent.

	2000	2001	2002	2003	2004	2005	2006	2007
Sources*	1	1	1,2	3		4		4
Norway	19	31	33			60.3		66.8
Denmark			47.2			61.8		71.6
Germany			24			44.4		56.6
Portugal			14	19.6		29.2		38.3
Greece			11.7			23.9		32.1

*Data sources: 1: Norwegian eHealth Survey [6], 2: Eurobarometer [8], 3: Network Society in Portugal [9–11], and 4: eHealth Trends [4, 7].
